# Involvement of the Leaf-Specific Multidrug and Toxic Compound Extrusion (MATE) Transporter Nt-JAT2 in Vacuolar Sequestration of Nicotine in *Nicotiana tabacum*

**DOI:** 10.1371/journal.pone.0108789

**Published:** 2014-09-30

**Authors:** Nobukazu Shitan, Shota Minami, Masahiko Morita, Minaho Hayashida, Shingo Ito, Kojiro Takanashi, Hiroshi Omote, Yoshinori Moriyama, Akifumi Sugiyama, Alain Goossens, Masataka Moriyasu, Kazufumi Yazaki

**Affiliations:** 1 Department of Natural Medicinal Chemistry, Kobe Pharmaceutical University, Kobe, Hyogo, Japan; 2 Laboratory of Plant Gene Expression, Research Institute for Sustainable Humanosphere, Kyoto University, Kyoto, Japan; 3 Department of Membrane Biochemistry, Okayama University Graduate School of Medicine, Dentistry and Pharmaceutical Sciences, Okayama, Japan; 4 Department of Plant Systems Biology, VIB, Gent, Belgium; 5 Department of Plant Biotechnology and Bioinformatics, Ghent University, Gent, Belgium; University of Cambridge, United Kingdom

## Abstract

Alkaloids play a key role in higher plant defense against pathogens and herbivores. Following its biosynthesis in root tissues, nicotine, the major alkaloid of *Nicotiana* species, is translocated via xylem transport toward the accumulation sites, leaf vacuoles. Our transcriptome analysis of methyl jasmonate-treated tobacco BY-2 cells identified several multidrug and toxic compound extrusion (MATE) transporter genes. In this study, we characterized a MATE gene, *Nicotiana tabacum jasmonate-inducible alkaloid transporter 2* (*Nt-JAT2*), which encodes a protein that has 32% amino acid identity with Nt-JAT1. *Nt-JAT2* mRNA is expressed at a very low steady state level in whole plants, but is rapidly upregulated by methyl jasmonate treatment in a leaf-specific manner. To characterize the function of Nt-JAT2, yeast cells were used as the host organism in a cellular transport assay. Nt-JAT2 was localized at the plasma membrane in yeast cells. When incubated in nicotine-containing medium, the nicotine content in Nt-JAT2-expressing cells was significantly lower than in control yeast. Nt-JAT2-expressing cells also showed lower content of other alkaloids like anabasine and anatabine, but not of flavonoids, suggesting that Nt-JAT2 transports various alkaloids including nicotine. Fluorescence assays in BY-2 cells showed that Nt-JAT2-GFP was localized to the tonoplast. These findings indicate that Nt-JAT2 is involved in nicotine sequestration in leaf vacuoles following the translocation of nicotine from root tissues.

## Introduction

As sessile organisms, higher plants have evolved various strategies to adapt to their environment. One important adaptation mechanism is to synthesize a large number of secondary metabolites, also called specialized metabolites, which are involved in protecting plants from environmental stresses. Alkaloids, a group of secondary metabolites involved in defending plants against pathogens and herbivores [1], have various biological activities, with some showing strong cytotoxicity. Some of these alkaloids are used as medicines, for example as anticancer drugs and analgesics. Alkaloids usually accumulate in a particular organelle of a specific organ or are excreted from cells, with some alkaloids transported from a source to a sink organ [2]. These findings suggest that plants have various transport systems, likely involving transporter proteins, which play important roles in the efficient production and accumulation of secondary metabolites.

Alkaloid transport mechanisms can be roughly divided into three types: transporter-independent trapping, vesicle-mediated transport, and transporter-mediated membrane transport [3]. Recently several alkaloid transporters have been isolated and characterized. These include CjABCB1/CjMDR1 (*Coptis japonica* ABCB1) and CjABCB2, which belong to the B-type ATP-binding cassette (ABC) transporter family and are involved in berberine uptake at the plasma membrane of rhizome cells and the accumulation of this alkaloid in *C. japonica* rhizomes [4], [5]. Another such protein is CrTPT2, a G-type ABC transporter of *Catharanthus roseus* that localizes to the plasma membrane of leaf cells and transports the indole alkaloid catharanthine to the leaf surface [6]. In addition, vacuolar transport in both *C. japonica* and *C. roseus* is thought to involve a proton/alkaloid transport system [7], [8]. However, our knowledge of the membrane transport mechanism of alkaloids is still limited.

To further identify and characterize alkaloid transporters, *Nicotiana tabacum* and its main alkaloid nicotine were used as a model to isolate transporters of the endogenous alkaloid. In *Nicotiana* species, nicotine, a pyridine alkaloid, is produced as the major secondary metabolite. When tobacco plants are attacked by insects or wounded, jasmonate-mediated signaling induces nicotine biosynthesis in the roots, the specific site of expression of nicotine biosynthetic genes [9]. The nicotine is then translocated to the aerial parts of the plant via xylem [10], resulting in the accumulation of nicotine in leaf vacuoles [11]. Whereas the concentration of free nicotine in xylem is about 1 mM, its concentration in vacuoles of epidermal cells at the tip of the leaf may be as high as 60 mM [12]. Because nicotine is strongly toxic to the nervous system of insects, this alkaloid functions as a defensive toxin against herbivores [13]. Thus, the translocation of nicotine from the roots to the leaves is highly important in tobacco defenses, and the existence of several transporters has been postulated.

To isolate nicotine transporters, cDNA-amplified fragment length polymorphism-based transcript profiling (cDNA-AFLP) was performed using Bright Yellow-2 (BY-2) cultured tobacco cells treated with methyl jasmonate (MeJA) [14]. This analysis enabled us to identify several multidrug and toxic compound extrusion (MATE) transporters, among which *Nicotiana tabacum* jasmonate-inducible alkaloid transporter 1 (Nt-JAT1) was characterized [15]. *Nt-JAT1* mRNA is expressed throughout the entire plant, including the leaves. Its gene product localizes to the tonoplast of leaf cells and transports nicotine and other alkaloids in a proton gradient-dependent manner. These data suggested that Nt-JAT1 plays an important role in nicotine translocation by acting as a proton antiporter, at least in leaves, responsible for unloading and deposition of alkaloids in the vacuoles [15]. Other tobacco MATE transporters, *NtMATE1* and *NtMATE2*, were isolated as down-regulated genes in roots of *nic1nic2* regulatory mutant, in which the accumulation of nicotine is lower than in wild-type tobacco plants [16]. NtMATE1 and NtMATE2 are specifically expressed in roots and localized to the tonoplast. These root-specific MATE proteins are thought to transport tobacco alkaloids into the vacuoles from the cytosol in alkaloid-synthesizing root cells [16]. In addition to MATE transporters, another type of nicotine transporter has also been recently identified. Nicotine uptake permease1 (NUP1), belonging to the purine uptake permease (PUP) transporter family, is localized to the plasma membrane and is involved in the movement of apoplastic nicotine into the cytoplasm of tobacco root cells, a process that affects nicotine metabolism and root growth [17]. However, overall knowledge about nicotine translocation in the plant body and the responsible transporters is still incomplete.

This study reports the characterization of a new MATE transporter, Nt-JAT2, which was identified by cDNA-AFLP analysis. We performed transcription analysis following treatment with plant hormones and stresses, transport assays using a heterologous expression system, and determined the subcellular localization of Nt-JAT2 protein in tobacco cells. The possible physiological function of this transporter in plant defense mechanisms is also discussed.

## Results

### Cloning and Sequence Analysis of *Nt-JAT2*

**Figure 1:**
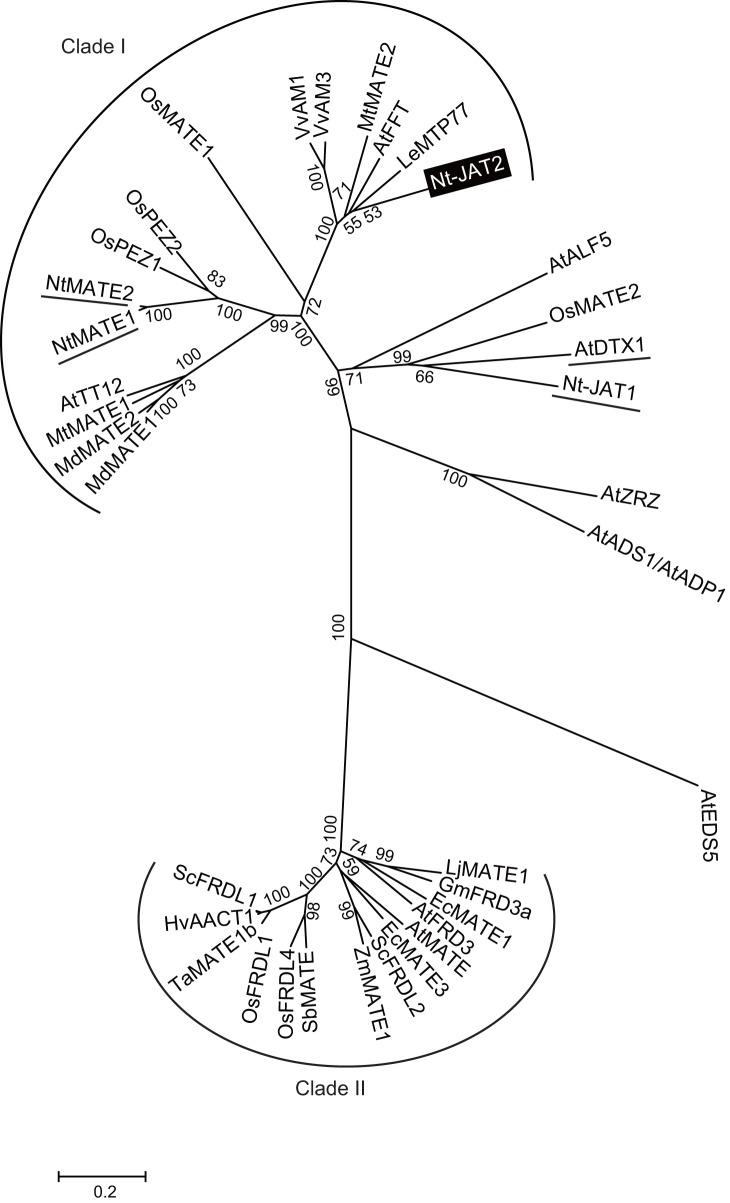
Phylogenetic relationship of plant MATE family members. Amino acid sequences of MATE members were aligned with ClustalW and subjected to phylogenetic analysis using MEGA6 software [43] with the neighbor-joining algorithm. Numbers on the branches represent bootstrap values. Proteins that transport alkaloids as substrates are underlined. The scale bar shows the number of amino acid substitutions per site. Most proteins belonging to clade I transport secondary metabolites, such as epicatechin 3′-*O*-glucoside, apigenin 7-*O*-glucoside, malvidin 3-*p*-coumaroylglucoside, nicotine, and protocatechuic acid; whereas almost all Clade II proteins transport citrate. Arabidopsis MATE members are: AtADS1/AtADP1, At4g29140; AtALF5, At3g23560; AtDTX1, At2g04070; AtEDS5, At4g39030; AtFFT, At4g25640; AtFRD3, At3g08040; AtMATE, At1g51340; AtTT12, At3g59030; AtZRZ/AtBCD1, and At1g58340. MATE members of other plant species and their accession numbers are: EcMATE1 (*Eucalyptus camaldulensis*), BAM68465; EcMATE3, BAM68467; GmFRD3a (*Glycine max*), ACE89001; HvAACT1 (barley), BAF75822; LeMTP77 (tomato), AAQ55183; LjMATE1 (*Lotus japonicus*), BAN59993; MdMATE1 (*Malus domestica*), ADO22710; MdMATE2, ADO22712; MtMATE1 (*Medicago truncatula*), ACX37118; MtMATE2, ADV04045; Nt-JAT1 (*Nicotiana tabacum*), CAQ51477; NtMATE1, BAF47751; NtMATE2, BAF47752; OsFRDL1 (*Oryza sativa*), BAG95121; OsFRDL4, BAL41687; OsMATE1, Os03g08900; OsMATE2, Os05g48040; OsPEZ1, AK243209; OsPEZ2, Os03g0572900; SbMATE (*Sorghum bicolor*), ABS89149; ScFRDL1 (rye), BAJ61741; ScFRDL2, BAJ61742; TaMATE1b (*Triticum aestivum*), AFZ61900; VvAM1 (*Vitis vinifera*), ACN91542; VvAM3, ACN88706; and ZmMATE1 (*Zea mays*), ACM47309.

In attempting to identify nicotine transporters in tobacco BY-2 cells using cDNA-AFLP analysis of jasmonate-inducible genes [14], we identified a new MATE transporter gene (clone No. C215, Fig. 1 of [15]), which was coregulated with alkaloid biosynthesis genes. The full-length cDNA of this gene was isolated by RT-PCR and 5′- and 3′-RACE. This full-length cDNA, designated *Nicotiana tabacum jasmonate-inducible alkaloid transporter 2* (*Nt-JAT2*), was approximately 1.8 kb long and encoded a putative polypeptide consisting of 507 amino acids. The Phobius prediction program for transmembrane regions (http://phobius.sbc.su.se/) suggested that Nt-JAT2 protein contains 12 transmembrane helices, with a topology representative of MATE transporters. Nt-JAT2 showed 32% amino acid identity with Nt-JAT1 and 38% identity with NtMATE1. Plant MATE transporters have been reported to transport various substrates, including flavonoids, alkaloids, citrate, and xenobiotics [18]. Phylogenetic analysis of the relationships between Nt-JAT2 and other plant MATE transporters showed that Nt-JAT2 belongs to clade I, with most proteins of the proteins in this clade being transporters of secondary metabolites as substrate (Figure 1). Nt-JAT2 showed 69% amino acid similarity with the protein encoded by tomato *MTP77*, a gene regulated by MYB transcription factor although its function has not yet been characterized [19]. Nt-JAT2 also showed 65% amino acid similarity with Arabidopsis flower flavonoid transporter (AtFFT), the substrate of which has not been identified, although a T-DNA knockout mutant showed altered flavonoid metabolism [20]. Nt-JAT2 was also found to be 61% similar at the amino acid level with VvAM1, VvAM3 and MtMATE2, which are involved in the vacuolar accumulation of anthocyanins in *Vitis vinifera* and *Medicago truncatula*, respectively [21], [22].

### Expression Analysis of *Nt-JAT2*

**Figure 2:**
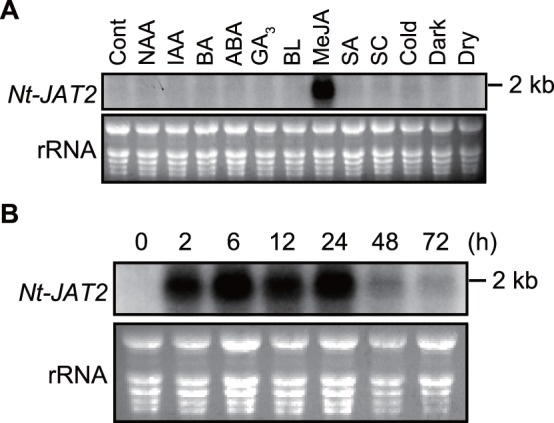
MeJA induction of *Nt-JAT2* mRNA expression in tobacco seedlings. (A) *Nt-JAT2* expression in response to various treatments. Fourteen-day-old seedlings were treated for 24 h with 10 µM 1-naphthaleneacetic acid (NAA), 10 µM IAA, 10 µM 6-benzyladenine (BA), 10 µM abscisic acid (ABA), 10 µM gibberellic acid (GA_3_), 5 µM brassinolide (BL), 100 µM MeJA, 100 µM salicylic acid (SA), or 100 µM sclareol (SC), at 4°C (cold)/low light, dark (dark), and drought (dry) conditions. Cont., untreated control. Total RNA (7.5 µg) prepared from the aerial parts of seedlings was probed with a ^32^P-labeled *Nt-JAT2* fragment (top). Loading controls are shown as EtBr-stained 18S rRNA (bottom). (B) RNA gel blot analysis of *Nt-JAT2* in tobacco seedlings. Seedlings were harvested 0 to 72 h after MeJA treatment. Total RNA (7.5 µg) was probed with a ^32^P-labeled *Nt-JAT2* fragment (0.5 kb) (top). Loading control is shown as EtBr-stained 18S rRNA (bottom). For comparison between NtJAT1 and NtJAT2, expression analyses were performed using the same membrane as our previous study [15].

**Figure 3:**
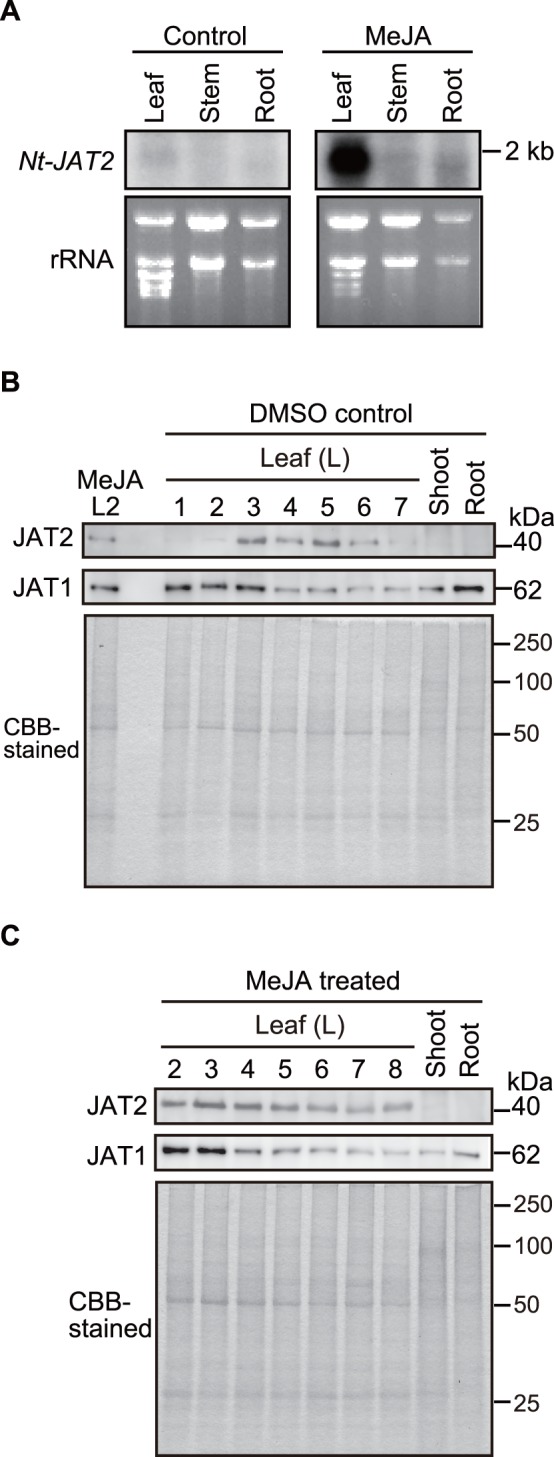
Expression of Nt-JAT2 in tobacco plants. (A) Organ-specific expression of *Nt-JAT2* mRNA in tobacco plants. Total RNA (7.5 µg) prepared from each tobacco organ was probed with ^32^P-labeled *Nt-JAT2* fragment (0.5 kb) (top). The amount of total RNA applied to each lane is shown by EtBr-stained 18S rRNA (bottom). For comparison between NtJAT1 and NtJAT2 expression, analysis was performed using the same membrane as our previous study [15]. (B, C) Immunoblot analysis of Nt-JAT2 and Nt-JAT1 proteins in control (B) and MeJA treated (C) plants. Microsomes from tobacco leaves, stems and roots were subjected to electrophoresis, blotted, and incubated with antibody to Nt-JAT2 or Nt-JAT1. L, leaves (Leaves were numbered from top to bottom).

To assess whether *Nt-JAT2* mRNA levels responded to various plant hormones, signaling molecules, and abiotic stresses, 14-day-old seedlings were treated with these agents, and RNA gel blot analysis was performed using an *Nt-JAT2-*specific probe. The level of expression of *Nt-JAT2* mRNA was very low in the absence of treatment and was little affected by almost all agents. Interestingly, however, MeJA, which strongly induces nicotine production in tobacco, specifically and strongly stimulated *Nt-JAT2* mRNA expression (Figure 2A). The expression profile of *Nt-JAT2* was clearly different from that of *Nt-JAT1*, which is expressed under control conditions and is induced by various treatments [15]. Furthermore, a time course experiment showed that *Nt-JAT2* mRNA expression increased markedly 2 h after MeJA treatment, with the high mRNA level maintained for 24 h (Figure 2B). Expression analysis of axenic adult plants grown in a culture vessel found that the expression of *Nt-JAT2* mRNA was low in leaves, stems, and roots, but was markedly enhanced by MeJA treatment, preferentially in leaves (Figure 3A).

We also analyzed the expression of Nt-JAT2 protein in plants. Immunoblot analyses showed that, under normal growth conditions, this protein was expressed only in the middle leaves (3rd to 6th leaves from top to bottom), whereas Nt-JAT1 protein expression was observed ubiquitously in leaves, stems and roots (Figure 3B). Upon treatment with MeJA, however, Nt-JAT2 expression was induced both in upper and lower leaves (Figure 3C).

### Nt-JAT2 Functions as a Nicotine Transporter

**Figure 4:**
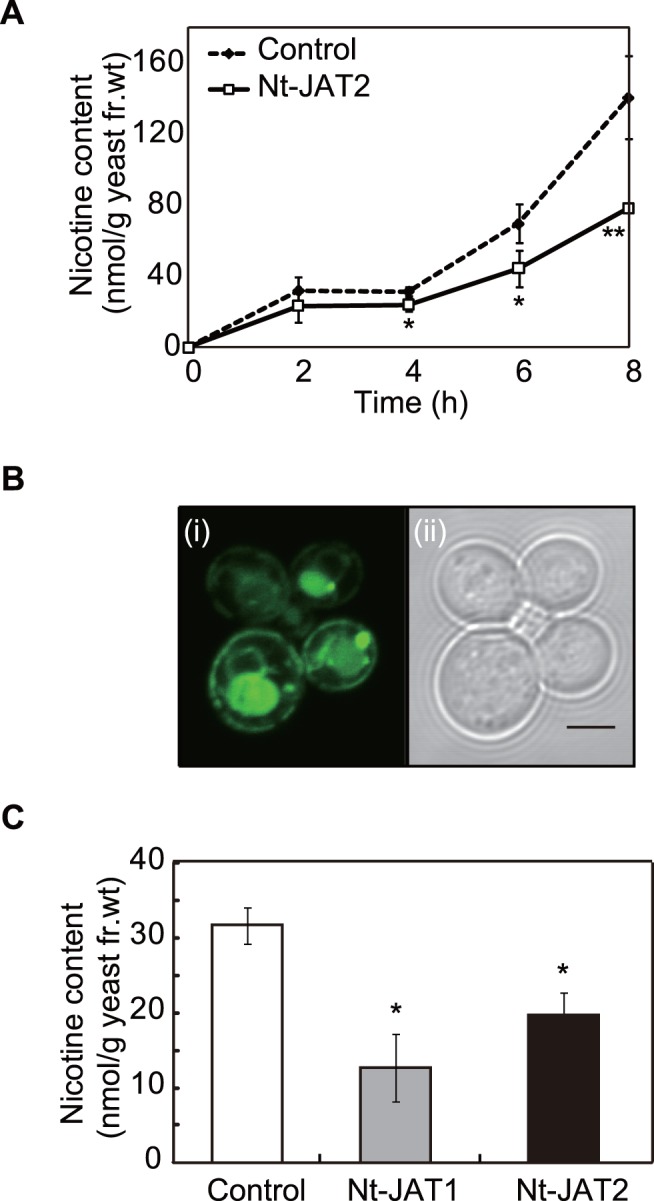
Nicotine transport activity of Nt-JAT2. (A) Time course analysis of nicotine transport in Nt-JAT2-expressing yeast. Control (dashed line) and Nt-JAT2-expressing (solid line) yeast cells were incubated in half-strength SD medium supplemented with 1 mM nicotine and sampled at the times indicated. Results are mean ± SD of triplicates. *P<0.05; **P<0.01 compared with control by Student’s t-test. (B) Localization of Nt-JAT2–GFP in yeast cells. Yeast cells expressing Nt-JAT2–GFP were grown at 30°C to logarithmic growth phase and observed by fluorescence microscopy. (i) Fluorescence of yeast cells transformed with Nt-JAT2–GFP; (ii) bright-field contrast (scale bar, 5 µm). (C) Nicotine content in control (white bar), Nt-JAT1-expressing (gray bar) and Nt-JAT2 -expressing (black bar) yeast cells incubated in half-strength SD medium containing 0.5 mM nicotine for 6 h. Results are mean ± SD of triplicates. *P<0.01 compared with control by Student’s t-test.

The transport activity of Nt-JAT2 was analyzed using a yeast cellular transport system [23]. Nt-JAT2 was expressed under the control of the constitutive promoter PMA1 in the yeast strain AD12345678, a strain that lacks several genes that encode ABC transporters conferring yeast multidrug resistance [24]. Control cells, transformed with the empty vector, and Nt-JAT2 transformant cells were incubated in nicotine-containing half-strength Synthetic Dextrose (SD) medium, and the intracellular nicotine content was quantitatively analyzed by HPLC. A time course analysis (from 2 to 8 h) showed that the nicotine content was consistently and significantly lower in Nt-JAT2 transformants than in control cells (Figure 4A). Fluorescence of Nt-JAT2-GFP protein was observed at the plasma membrane of yeast cells, as well as in the vacuolar lumen (Figure 4B). Because Nt-JAT2 is a membrane protein, fluorescence in the vacuolar lumen was unexpected, but it may be due to GFP that had been cleaved from Nt-JAT2-GFP mislocalized to tonoplasts and digested by yeast endogenous proteases (Figure S1). Nevertheless, ca. 40% of Nt-JAT2 localized to the plasma membrane and functioned as a nicotine efflux transporter in yeast cells presumably using proton gradient across the plasma membrane, reducing the nicotine content in Nt-JAT2 transformants. The cellular nicotine content of Nt-JAT2 was similar to that of Nt-JAT1 (Figure 4C), suggesting that these transporters have the same level of nicotine efflux activity.

**Figure 5:**
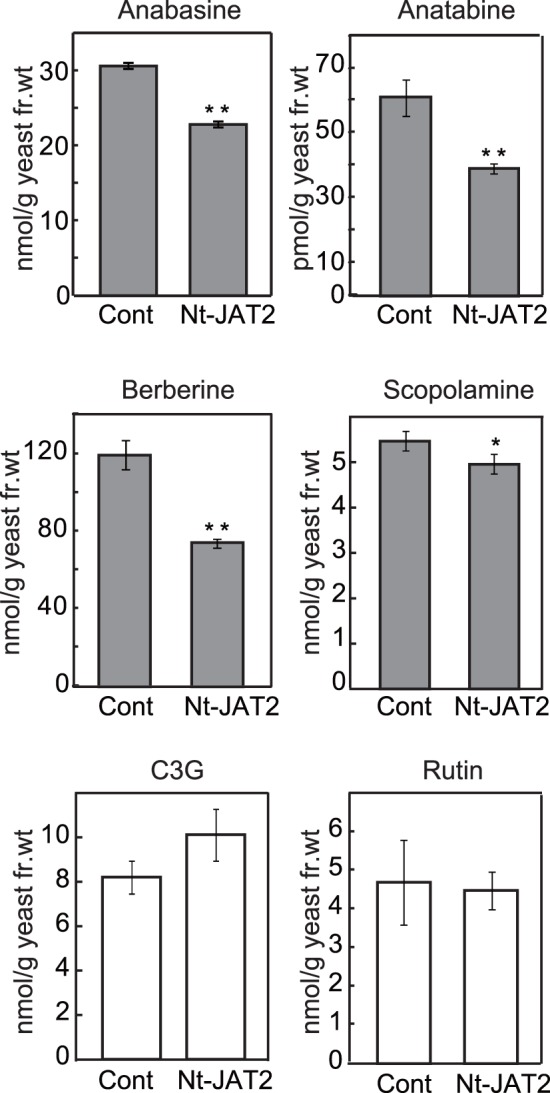
Substrate specificity of Nt-JAT2. Yeast cells were incubated in half-strength SD medium containing anabasine (50 µM), anatabine (50 µM), berberine (50 µM), scopolamine (50 µM), C3G (75 µM), or rutin (50 µM) for 6 h. The cells were collected by centrifugation, and the cellular content of each substance was quantitatively analyzed. Results are mean ± SD of triplicates. *P<0.05; **P<0.01 compared with control by Student’s t-test.

We also analyzed the substrate specificity of Nt-JAT2 using various natural compounds (Figure 5 and Figure S2). Treatment of yeast cells with other tobacco alkaloids, such as anabasine and anatabine, significantly reduced the cellular content of Nt-JAT2 to a lower level than in control cells. Nt-JAT2 also recognized the plant alkaloids berberine and scopolamine as transport substrates. Nt-JAT2-expressing yeast cells were also incubated with the flavonoid glycosides, cyanidin 3-*O* glucoside (C3G), a substrate of *M. truncatula* MATE1 (MtMATE1), MtMATE2 and Arabidopsis TT12, and rutin [22], [25], [26]. In contrast to the alkaloids, the cellular contents of C3G and rutin did not differ significantly between control and Nt-JAT2 transformant cells, suggesting that Nt-JAT2 does not recognize these flavonoids as substrates (Figure 5). Since kaempferol is a putative substrate of OsMATE1, we also tested two flavonoid aglycons, quercetin and kaempferol [27], but neither affected nicotine transport by Nt-JAT2 (Figure S3). These results suggested that Nt-JAT2 recognizes alkaloids but not flavonoids as substrates.

### Nt-JAT2 is Localized to the Tonoplast in Tobacco Cells

**Figure 6:**
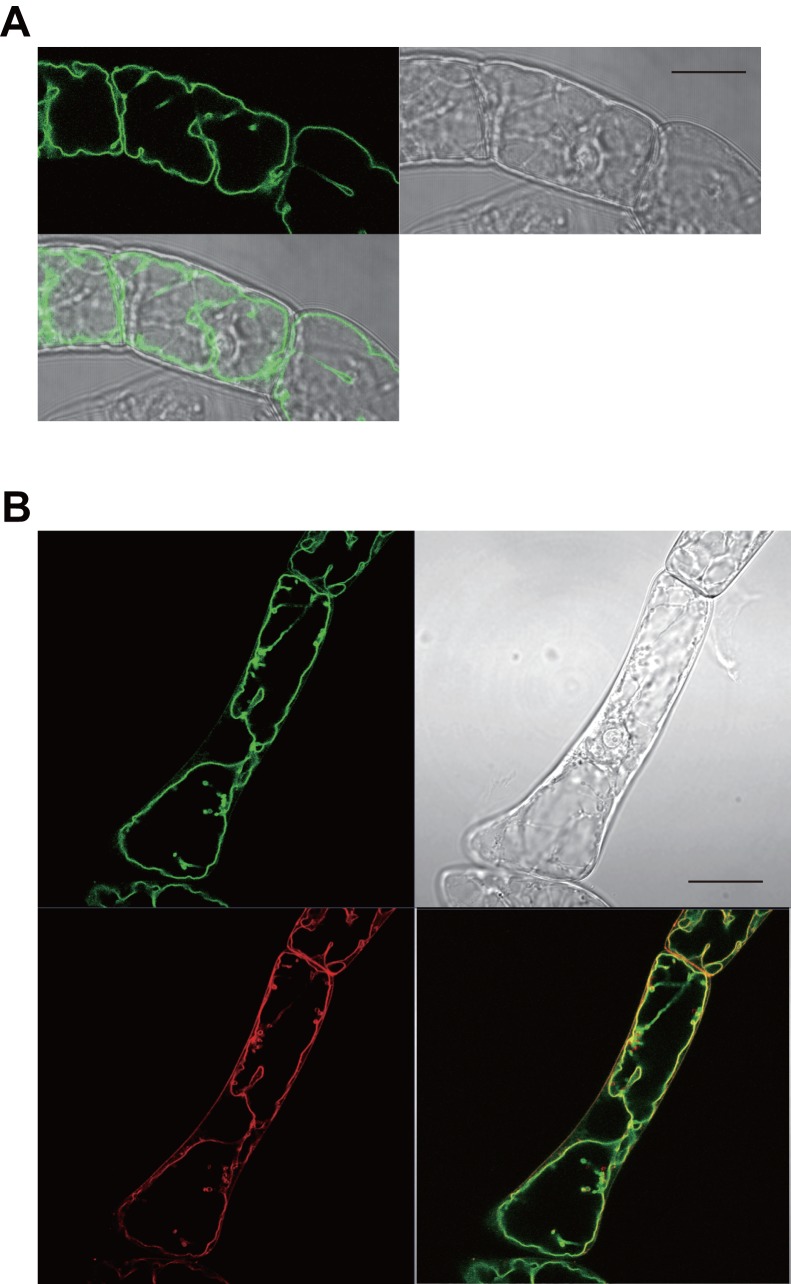
Subcellular localization of Nt-JAT2 in cultured tobacco cells. (A) Confocal images of Nt-JAT2-GFP in transgenic BY-2 cells. (left) Nt-JAT2-GFP fluorescence images, (right) bright field images, (bottom left) merged images. (B) Nt-JAT2-GFP fluorescence (green, top left), FM4-64 fluorescence (red, bottom left), merged (bottom right), and bright field (top right) images after treatment for 24 h (scale bar, 2 µm.).

Our preliminary experiments suggested that Nt-JAT2 in tobacco cells localizes to tonoplasts [28]. To investigate the subcellular localization of Nt-JAT2 more precisely, we expressed Nt-JAT2-GFP protein under the control of the cauliflower mosaic virus 35S promoter in tobacco BY-2 cells. Fluorescence analysis showed that Nt-JAT2 clearly localized to the tonoplast with high reproducibility (Figure 6A). To confirm the tonoplast localization of Nt-JAT2, we used the endocytic tracer dye FM4-64, which stains mainly tonoplast after long incubation [29]. Following staining with FM4-64 for 24 h, we found that the fluorescence of Nt-JAT2-GFP (green) and FM4-64 (red) overlapped (Figure 6B), indicating that Nt-JAT2 localizes to the tonoplast in tobacco cells. Taken together, our results suggest that Nt-JAT2 participates in nicotine accumulation in leaf vacuoles by acting as a nicotine transporter.

## Discussion

### Physiological Function of Nt-JAT2

This study was designed to assess the function of a novel MATE transporter, Nt-JAT2, involved in secondary metabolite transport. Functional and expression analyses of Nt-JAT2 suggested that Nt-JAT2 may be involved in the translocation of nicotine into the leaves of tobacco plants. Nicotine is biosynthesized in the root tissues of tobacco plants and is transported to the leaves via xylem. Once arriving in the leaves, nicotine is transported by tonoplast-localized Nt-JAT2 to the vacuolar lumen of the leaves, where it accumulates. When tobacco plants are attacked by herbivores, Nt-JAT2, which is induced in younger and older leaves, would contribute to optimal distribution of limited amounts of nicotine to these vulnerable parts, leading to the protection of such tissue. As Nt-JAT1 was also shown to act as an alkaloid transporter in leaves [15], these two MATE transporters may function cooperatively in the accumulation of alkaloid in leaf vacuoles. Therefore, Nt-JATs mediated sequestration of toxic alkaloids in leaves can protect these organs against herbivores.

### Allotment of MATE Transporters in Tobacco Plants

Plants have many transporters of different classes. Regardless of class, some transporters accept the same substrates with similar transport properties, however, their physiological functions differ according to the expressing tissue and/or localizing membranes [30]. It is to be noted that plant MATE transporters have different physiological functions in the transport of secondary metabolites, due to differences in their temporal and spatial expression patterns. For example, Nt-JAT1 is expressed not only in leaves but in stems and roots [15], NtMATE1 and NtMATE2 are expressed only in roots [16], and Nt-JAT2 is expressed almost exclusively in leaves, with its expression specifically and strongly induced by MeJA. These results suggest that Nt-JAT2 is specialized for the accumulation of nicotine and related alkaloids in leaves upon herbivore attack, whereas Nt-JAT1 is responsible for steady-state alkaloid transport and for transport in various tissues.

Nicotine plays important roles not only in plant defense mechanisms but also in the rates of outcrossing and seed production through its activity as a nectar repellent [31]. Thus, other transporters may also be involved in nicotine translocation; e.g., plasma membrane efflux from root cells and plasma membrane influx into cells of leaves or flowers. Although Nt-JAT2-GFP overexpression under the control of a 35S promoter in tobacco plant stable transformants did not strongly affect the nicotine content in leaves (data not shown), identification of other alkaloid transporters, in combination with Nt-JAT2, may enable the nicotine content to be altered efficiently in tobacco plants by transport engineering. Candidates for such transporters included PUP, ABC, nitrate and peptide transport (NRT/PTR) and other MATE transporters [3], [18], [32], [33]. Further investigation of such transporters will clarify the entire mechanism of nicotine translocation in tobacco plants.

### MATE Proteins as Secondary Metabolite Transporters

Plant genomes contain many MATE transporter genes; e.g., 56 in *Arabidopsis thaliana* [18]. Some of these genes encode proteins, such as AtDTX1 and ALF5, which recognize various substrates and are involved in detoxification mechanisms [34], [35]. In addition, a bacterial MATE transporter was originally identified as a multidrug efflux transporter [36], whereas, some MATE family members, in particular plant MATE transporters of clade II (Figure 1), have a strict specificity for endogenous substrates. AtFRD3 and its homologous proteins, such as LjMATE1, transport citrate with high substrate specificity [37], [38]. Furthermore, MATE proteins of clade I have been reported involved in the transport of various secondary metabolites [3], [18], [39]. In tobacco plants, several MATE transporters including Nt-JAT2 have been reported in alkaloid transport. Thus, MATE transporters are a major family responsible for the membrane transport of secondary metabolites in plant bodies.

### Conclusion

A novel MATE transporter, Nt-JAT2, was isolated from *N. tabacum* using transcriptome analysis (Figure1). *Nt-JAT2* was specifically expressed in leaves, where nicotine accumulates to high levels. In addition, *Nt-JAT2* was strongly induced by MeJA in a specific manner, which induces nicotine biosynthesis as well (Figure 2, 3). This protein is localized to the tonoplast in tobacco, and function as nicotine efflux transporter with moderate substrate specificities to alkaloids (Figure 4–6). These data suggest that Nt-JAT2 is responsible for transporting nicotine into the vacuole of leaves and is involved in nicotine translocation and plant defense mechanism in tobacco. Transport process of secondary metabolites is a newly developing research area. Our findings add new knowledge to this field, and will further contribute to understand overall scheme of the movement and accumulation mechanism of secondary metabolites in plants.

## Materials and Methods

### Chemicals

Nicotine and other chemicals used in this study were purchased from Wako Pure Chemicals (Osaka, Japan) or Nacalai Tesque (Kyoto, Japan).

### Plant Materials

*N. tabacum* L. (cv. Samsun NN) plants were grown under 16-h light/8-h dark cycles at 25°C. Cultured cells of *N. tabacum* L. cv. BY-2 were maintained in suspension as described [40]. To analyze the regulation of *Nt-JAT2* transcripts, seeds were sown on nylon mesh (6×6 cm, 200-µm pore; CellMicroSieves, BioDesign, New York, U.S.A.) over half-strength Linsmaier and Skoog (LS) medium (1.5% sucrose, 0.8% agar, pH 5.8) and grown for 14 days under the same light/dark regime as described above. Roots were subjected to various treatments by gentle transfer of the mesh to new medium. After 24 h, treatments were stopped by immediate freezing of seedlings in liquid N_2_. To analyze the tissue-specific expression of *Nt-JAT2* mRNA in tobacco plants, four sterilized pieces of filter paper soaked with 1.25 ml of a 500 µM MeJA solution were placed beside the 2-month-old plant in a tightly sealed Agripot (Kirin, Tokyo, Japan) for 24 h.

### Cloning of Full-length cDNA, RNA Isolation and Expression Analysis

A full-length cDNA clone of tobacco MATE *Nt-JAT2* (GenBank/EMBL/DDBJ accession no. AB922128) was obtained by the combination of PCR amplification from a cDNA library of MeJA-treated tobacco BY-2 cells and 5′ RACE using GeneRacer (Invitrogen, Carldsbad, CA, U.S.A). *Nt-JAT2*-specific primers used for the library screening were designed based on the cDNA-AFLP tag sequence obtained previously [14] (GenBank/EMBL/DDBJ accession no. CQ808789): Nt-JAT2P-forward (fw), 5′-GATCCCTCTCTATTTGCAT-3′; Nt-JAT2P-reverse (rev) 5′-CCTACAAGCCAAGCCAGGT-3′.

Gene-specific primers used for 5′-RACE were Nt-JAT2_RACE_1^st^, 5′-GAGAGTCTCCAGATCACTCCCCATGC-3′; and Nt-JAT2_RACE_2^nd^, 5′-TGTAGCTCCAAGATGGCCGCAGAAAG-3′.

RNA isolation and expression analysis were performed as described previously [15]. Hybridization was performed with an *Nt-JAT2* specific probe (positions +363 to +807).

### Antibody Against Nt-JAT2 Peptide

An oligopeptide of Nt-JAT2, corresponding to amino acids at positions 17–32 (n-CLIGPDGDYRRIRGLKE-c), was conjugated to keyhole limpet hemocyanin conjugate, and injected into a rabbit using a standard protocol (Hayashi Kasei, Osaka, Japan). After the third boost, the antiserum was recovered and used for immunoblot analysis against Nt-JAT2 without further purification. Antibodies against Nt-JAT1 were prepared and used as described [15]. Specificity of Nt-JAT1- or Nt-JAT2- antibodies was confirmed by using heterologously expressed Nt-JAT1- or Nt-JAT2-proteins, respectively (data not shown).

### Detailed analysis of Nt-JAT2 Expression in Leaves

To analyze the expression of Nt-JAT2 protein, a small piece of cotton soaked with 109 µl of a 458 µM MeJA solution, was placed beside the 3-week-old plant for 24 h. For immunoblotting, extracted membrane proteins were denatured in denaturation buffer for 10 min at 50°C, subjected to SDS-PAGE, and transferred to Immobilon poly(vinylidene difluoride) membranes (Millipore, Tokyo, Japan). The membrane were treated with BlockingOne (Nacalai Tesque) to block nonspecific binding, and incubated with primary antibodies (Nt-JAT1, 1∶500 dilution; Nt-JAT2, 1∶1000 dilution) and secondary horseradish peroxidase-conjugated anti-rabbit IgG antibodies (1∶10000 dilution; GE Healthcare, UK) using standard procedures. The band was visualized by Chemi-Lumi One Super (Nacalai Tesque).

### Vector Construction and Transformation

The *Nt-JAT2* coding sequence was amplified with Phusion DNA polymerase (Thermo Fisher Scientific, Waltham, MA, U.S.A.) using the primer pair Nt-JAT2_Fw_*Eco*RI, 5′- CGGAATTCATGGAATCACCATTGCTG -3′; and Nt-JAT2_-stop_*Not*I, 5′- AAGGAAAAAAGCGGCCGCGACGCGTAAATCTTAGCTCTT -3′, where the underlined sequences indicate non-native *Eco*RI and *Not*I sites for subcloning, respectively. The PCR product was subcloned into pENTR1A (Invitrogen), and the *Nt-JAT2* cDNA fragment was excised and cloned into the site between the 35S promoter and GFP coding region of the binary vector pGWB5 using the LR reaction (Invitrogen). The resulting construct, pGWB5-Nt-JAT2 was introduced into *Agrobacterium tumefaciens* strain LBA4404. Tobacco BY-2 cells were transformed with the binary vector.

### Subcellular Localization of Nt-JAT2 in Tobacco and Yeast

To visualize the tonoplast with the fluorescent probe FM4-64 (Molecular Probes Inc., Eugene, OR, U.S.A.), Nt-JAT2-GFP-expressing tobacco BY-2 cells were incubated with 8 µM FM4-64 for 24 h at 25°C, and monitored for red fluorescence. The green fluorescence of the expressed Nt-JAT2-GFP protein was visualized with a Zeiss LSM700 confocal microscope. The fluorescence of GFP and FM4-64 were detected by 488/490–555 nm and 555/640 excitation/emission filter sets, respectively.

Nt-JAT2 was subcellularly localized in yeast cells using an Nt-JAT2-GFP fusion construct. The cDNA fragments carrying the coding regions of *Nt-JAT2* and *GFP* were separately amplified, fused by crossover PCR and subcloned into the yeast expression vector pDR196. The fluorescence of Nt-JAT2-GFP was visualized with a Zeiss LSM700 confocal microscope (Carl Zeiss, Oberkochen, Germany) and the subcellular location of this protein in yeast cells was determined.

### Functional Analysis of Nt-JAT2 in Yeast

Stock solutions of transport substrates were prepared as follows: nicotine, anabasine, and anatabine were dissolved in EtOH; berberine and scopolamine were dissolved in water; and rutin, quercetin, kaempferol, and C3G were dissolved in DMSO. For yeast cellular transport assays, alkaloids and rutin were added at final concentration of 50 µM. C3G was added at 75 µM. For the inhibitor treatments, nicotine, quercetin, or kaempferol was added at 5 µM, due to the insolubility of flavonoids in water.

*Nt-JAT2* cDNA was subcloned into a yeast expression vector pDR196 with a strong constitutive promoter PMA1 [41]. The resulting plasmid, pDR-Nt-JAT2, was used to transform yeast strain AD12345678 (*MATα, PDR1-3, ura3, his1, Δyor1::hisG*, *Δsnq2::hisG*, *Δpdr5::hisG*, *Δpdr10::hisG*, *Δpdr11::hisG*, *Δycf1::hisG*, *Δpdr3::hisG;* and *Δpdr15::hisG*) [24] by the lithium acetate method, with transformants selected using SD medium (-uracil). Yeast transformants were treated as described [23]. Briefly, transformants were precultured in 40 mL of SD medium (-uracil), harvested at OD_600_ = 1.0, and suspended in 40 mL half-strength SD medium (-uracil) containing compound at OD_600_ = 1.0. The cells were incubated at 30°C with shaking at 200 rpm, harvested at the indicated time by centrifugation, washed twice with deionized cold water, and disrupted with glass beads and extraction buffer. Extraction buffers consisted of 50% ethanol, 50% methanol, 0.5% acetic acid for nicotine, anabasine, and anatabine; 50% methanol (0.05 N HCl) for berberine and C3G; 50% methanol (0.05 N H_2_SO_4_) for scopolamine; and 50% ethanol for rutin. The samples were centrifuged and the supernatants were subjected to HPLC or LC-MS analysis.

### HPLC Analysis

Nicotine, anabasine, anatabine, and berberine were quantified as described [4], [15], [42]. C3G and rutin were separated on a Cosmosil 5C_18_ MS-II column (5 µm, 4.6 mm×150 mm, Nacalai Tesque). Compounds were eluted at 1.0 ml/min with a solvent system consisting of (A) water/formic acid (9/1, v/v) and (B) water/formic acid/MeOH/acetonitrile (40/10/22.5/22.5, v/v/v/v), with a linear gradient program from 12% to 100% (B) in 15 min. C3G was detected at a wavelength of 516 nm, and rutin at 358 nm. Scopolamine was analyzed by LC-ESI-MS, using a Shimadzu HPLC system (Shimadzu, Kyoto, Japan) consisting of a binary pump (LC-10AD liquid chromatograph), an automatic solvent degasser (DGU-14A degasser), and an autosampler (SIL-10AD auto-injector) coupled to an API3000 LC-MS system (Applied Biosystems, Foster City, CA, U.S.A) equipped with an ESI interface. Scopolamine was separated on an XTerra column (5 µm, 4.6 mm×150 mm, Waters) using a mobile phase consisting of (A) 30% MeOH and (B) 70% (NH_4_)HCO_3_ buffer (10 mM, pH 9, adjusted with NH_4_OH) at a flow rate of 0.2 ml/min, in isocratic condition.

## Supporting Information

Figure S1 **Expression of Nt-JAT2-GFP protein in yeast cells.** Proteins were extracted from yeast cells (vector control, Nt-JAT2-GFP), subjected to SDS-PAGE, and transferred to Immobilon poly(vinylidene difluoride) membranes. The membrane were treated with BlockingOne, and incubated with primary antibodies (anti-GFP, 1∶1000 dilution; Nacalai Tesque) and secondary horseradish peroxidase-conjugated anti-mouse IgG antibodies (1∶10000 dilution; GE Healthcare, UK) using standard procedures. The band was visualized by Chemi-Lumi One Super (Nacalai Tesque).
(EPS)

Figure S2 **Chemical structures of alkaloids and flavonoids tested in this study.**
(EPS)

Figure S3 **Effects of flavonoids on nicotine transport in yeast cells.** Yeast cells were incubated with 5 µM nicotine in the absence (control) or presence of 5 µM quercetin or kaempferol. Results are mean ± SD of triplicates.
(EPS)
